# “A turn in the road, but still a rough journey” - Parent and child perspectives of outcomes after pre-adolescent inpatient psychiatric admission

**DOI:** 10.1186/s13034-023-00649-0

**Published:** 2023-09-02

**Authors:** Tania T Swart, Eugene L Davids, Petrus J de Vries

**Affiliations:** Division of Child & Adolescent Psychiatry, University of Cape Town, Cape Town, South Africa

**Keywords:** Child and adolescent mental health, Inpatient psychiatric admission, Mental health, Mental health outcomes, Inpatient unit, South Africa

## Abstract

**Background:**

Research regarding the outcomes of child and adolescent psychiatric inpatients appears insufficient and neglected. Where data are available, the majority of studies focus on adolescents. This study aimed (a) to describe child and parental perspectives of short-, medium- and long-term outcomes of children who have had a pre-adolescent inpatient psychiatric admission, and (b) to analyse these dyadic experiential data to identify outcome variables of importance to families and service users that could be used in future outcomes-based research.

**Methods:**

The study employed a qualitative methodology, using semi-structured in-depth interviews of ten parent-child dyads to (a) collect the perspectives of children who have had an inpatient psychiatric admission at a pre-adolescent unit and their parents, and (b) to analyse the experiences of inpatient admission and perceived outcomes after discharge using thematic analysis.

**Results:**

Overall, inpatient psychiatric admission was viewed as a positive and empowering experience by parents and children. Clear short-term benefits were reported as a result of new diagnoses, medications and new skills gained through the admission process. More than half of parent-child dyads reported long-term benefits, but many families commented on ongoing challenges. Thematic analysis identified diagnostic certainty, acquisition of cognitive and behavioural skills, appropriate educational environment, peer relationships, sustained follow-up and medication management, and parent-child relationships, as important contributors to outcomes. Importantly, the presence or lack of these elements influenced outcomes.

**Conclusions:**

The study explored parent-child dyadic perspectives about their experiences of inpatient mental health admissions for pre-adolescents and perceived outcomes after admission. The majority of families found inpatient admission positive and helpful, and thematic analysis identified a number of functional variables that may predict outcome. However, positive outcomes were associated with ongoing difficulties over time, as indicated by the theme “a turn in the road, but still a rough journey”.

## Background

Approximately 20% of children and adolescents have mental health disorders [1] and between 50 and 75% of adult mental illness commences before the age of 18 [2]. However, in most countries, fewer than 50% of children with mental health problems receive any treatment. In low- and middle-income countries (LMIC) this ‘treatment gap’ is often in excess of 90% [3–5]. Where services do exist for child and adolescent mental health (CAMH), countries rarely evaluate the outcome of treatments in a systematic way, leading to an ‘information gap’ [6]. Pedrini and colleagues [7] emphasised how this information gap could hinder clinical and research progress, given that knowledge of the outcomes and quality of care and interventions is needed (a) to ensure a high quality of care, (b) to ensure interventions are evidence-based, (c) to generate evidence for interventions where it does not exist, and (d) to provide data for health economic and policy development purposes.

Globally, information about inpatient pre-adolescent psychiatric units is limited. Young people may be admitted to inpatient psychiatric units for various reasons, including to receive detailed assessment or treatment away from the family home, in response to the escalation of symptoms despite intensive outpatient treatment, or to participate in controlled trials of specific interventions [8]. Inpatient admission comes with potential ‘risks’ and ‘benefits’ [8]. Most studies suggest that intensive outreach can reduce the need for inpatient admission but cannot replace it [8]. Interestingly, only half of high-income countries (HIC) have dedicated inpatient units for children with mental health disorders. In LMIC very few dedicated units for CAMH exist [6]. Across African countries, a survey of available services identified only six inpatient units that admitted preadolescent children [9]. One was in Uganda, two were in Egypt and three in South Africa (Weskoppies Child and Adolescent Unit in Pretoria, a Children’s Ward at Tara Psychiatric Hospital in Johannesburg, and the Therapeutic Learning Centre in Cape Town).

Research regarding the outcomes of child and adolescent psychiatric inpatients appears insufficient and neglected. Where data are available, the majority of studies to date have focussed on adolescents. As a result, knowledge about outcomes after admission to a pre-adolescent inpatient unit remains very limited. Past reviews [10–13] claimed inpatient admission to be largely beneficial. However, many of the studies were found to have had questionable methods and indistinct outcomes [11–13]. Previous studies present research challenges which include low patient numbers, inevitable patient variability, as well as the unique and highly variable characteristics of inpatient units [14]. Apart from these obvious methodological challenges, dynamic developmental considerations complicate any such research. For example, children may have fluctuating symptom profiles [10, 13], and the highly dynamic developmental stages of childhood need to be taken into account [11, 15]. Additionally, the unique and highly variable contextual factors and experiences of children admitted as inpatients have made systematic research on pre-adolescent inpatient outcomes difficult [15].

The contemporary body of research suggests the need for international consensus on key variables for the measurement of outcomes, identification of standardized outcome measures, and agreement on levels of outcomes to be explored (e.g. child, family, education, community). However, given the lack of current consensus on outcomes, we decided to go back to the ‘source’ of outcomes by listening to the voices of children and their caregivers [16] (a) to describe child and caregiver perspectives of short-, medium- and long-term outcomes of children who have had a pre-adolescent inpatient psychiatric admission, and (b) to analyse these dyadic experiential data to identify outcome variables of importance to families and service users. We proposed that such qualitative findings, based on the lived experience of young people and their families, could inform meaningful outcome measurement for future studies.

The current study, therefore, aimed to describe children who have had a pre-adolescent inpatient psychiatric admission and to explore their and their primary caregivers’ experiences and perceived short-, medium-, and long-term outcomes after discharge from an inpatient psychiatric unit in Cape Town, South Africa. The decision to include caregiver-child dyads was based on the importance of families in relation to the mental health of children.

## Methods

### Study design

The study used a qualitative descriptive approach (a) to describe parental and child perspectives of outcomes after pre-adolescent inpatient psychiatric admissions, and (b) to use these experiential data to identify themes that could indicate meaningful outcome variables for more formal outcomes-based research. The study was reported using the consolidated criteria for reporting qualitative research (COREQ) checklist.

### Recruitment

Following ethical approval from the University of Cape Town Faculty of Health Sciences Human Research Ethics Committee (HREC Ref 585/2018), permission to conduct the study was obtained from the clinical head of the inpatient unit in the Western Cape and the Research Review Committee of the Red Cross War Memorial Children’s Hospital (RXH: RCC169). All clinic registers and inpatient files for the years 2009–2017 were searched for the contact information of families. The inclusion criteria used when searching the clinic registers and inpatient files included: (i) the patient should have had an inpatient admission between the years of 2009–2017, (ii) the inpatient should be able to provide feedback or reflect on their experience while being an inpatient, (iii) the inpatient should not be residing outside of the Western Cape Province which might make attending the interview difficult and (iv) patient should not be in a children’s home as the study wanted to examine the experiences of parent-child dyads. Data were provided to the clinical head, who shared data with the primary clinical contacts of potential participants. Primary clinical contacts were the clinical team members who were actively working with proposed families or were the most recent clinical team members to have worked with families. Employing convenience sampling, eligible families were contacted telephonically by clinical team members to ask if they would be interested to participate in the study. Thirty-four families were approached, of which twenty-four families were reached. Families who indicated a willingness to participate were asked their contact preference for an invitation to the study. The primary researchers subsequently invited twelve of these families to participate, ten of whom accepted. Reasons for non-participation of contacted families included time constraints, ill-health, and not living in Cape Town anymore.

### Setting and data collection

All participants, both child inpatients and their parents, were given an information sheet, informed about the study, and invited to a face-to-face semi-structured individual interview (SSII) lasting approximately 40 min. Prior to free and voluntary participation, participants 18 years and older gave informed written consent, while those younger gave informed written assent. Interviews were conducted in a quiet, private room on the premises of the Division of Child & Adolescent Psychiatry, University of Cape Town. The interviews were conducted by a psychology research assistant who was female and had a Master’s degree in Psychology. She had experience working in a clinical setting and had training in clinical psychology. The research assistant had not been involved in any aspect of inpatient care of any of the participants but had an interest in the field of study. The research assistant informed participants of her independence from the inpatient clinic. Children and parents completed a demographic questionnaire and were interviewed separately. All interviews were conducted in English, which was indicated by all families as their home language and were audio-recorded with participant consent. An interview schedule with five open-ended questions was used to provide a basic structure to the SSII. The questions were shared with clinicians to reach a consensus on the appropriateness of the questions asked. The questions (child version) included (1) When you were at the (name of psychiatric unit), what things got better or worse for you?, (2) After leaving the (name of psychiatric unit), what things got better or worse for you?, (3) What helped or made things difficult for you after leaving the (name of psychiatric unit)?, (4) What was it like to be at the (name of psychiatric unit)?, (5) Are there some things that you remember about the (name of psychiatric unit) that you could tell me about? The research assistant kept brief notes after each interview. No other materials or measures were used and no repeat interviews were conducted. To ensure anonymity and confidentiality, all participants were allocated a number, and pseudonyms were used in the transcriptions. Families were given ZAR100 (~ US$7) as a ‘thank you’ for their participation.

### Data analysis

Audio recordings of the interviews were transcribed verbatim by an independent researcher and qualitative data were analysed using thematic analysis, as outlined in six phases by Braun and Clarke [17]: familiarisation with the data, generating initial codes, reviewing themes, defining and naming themes, and producing the report. No transcripts and codes were shared with participants for comment. The first author identified and coded generated categories [18] from all transcripts to identify emerging themes. A second author and coder, with high-level experience in qualitative analysis, independently coded 30% of the transcriptions for emerging themes. All coding was conducted manually without software by both coders. Identified codes and emerging themes were then revised and refined. The findings were interpreted within the context of existing literature on the outcomes of children after inpatient psychiatric admission. Given that all the available participants participated in the study, no additional recruitment took place and analysis continued until saturation was reached.

### Trustworthiness

The study performed thematic analysis of data which may be open to bias. To reduce potential bias and to increase trustworthiness, we (a) invited all families who had previous inpatient admission (to avoid pre-selecting those with positive experiences or outcomes), (b) ensured that interviews were conducted by a research assistant who had no link or association with the inpatient unit or staff, and (c) ensured that a senior researcher with no previous involvement in the inpatient unit independently was used to code 30% of transcripts.

### Reflexivity

Reflexivity is concerned with an active examination and reflection of judgements being made by the researchers in a study. Reflexivity was observed in the study through (i) the research assistant (independent to the inpatient unit, staff and families) conducting the interview keep brief notes and a reflective journal of thoughts, reflections and feeling both during and after the interview, (ii) two researchers (TTS and ELD) jointly performing thematic analysis – one researcher with previous working experience in the specific inpatient unit, the other a researcher with no previous clinical or research involvement in the setting.

### Ethics

The study adhered to the ethical principles of research. Research Ethics Clearance was provided by the University of Cape Town (HREC Ref 585/2018) and Red Cross War Memorial Children’s Hospital (RXH: RCC169). Informed consent/assent was obtained from all participants in the study.

## Results

### Demographic characteristics of participants

Participants consisted of a subset of all children who were inpatients at a pre-adolescent psychiatric unit in the Western Cape, South Africa between 2012 and 2017, and their parents. The final group of participants consisted of ten parent-child dyads and are shown in Table 1. Of these ten, two of the children were admitted as psychiatric inpatients in 2012 (n = 2; 20%), two in 2013 (n = 2; 20%), one in 2014 (n = 1; 10%), two in 2015 (n = 2; 20%), one in 2016 (n = 1; 10%) and two in 2017 (n = 2; 20%). The period after inpatient discharge ranged from 15 months to 8 years. The sample was predominantly males (n = 7; 70%) including one birth-assigned female who now identifies as male. Ages during inpatient admission ranged between 7 and 14 years (M = 10,5; SD = 2) with the majority in the 10-12-year-old range (n = 7; 70%). Age at the time of research participation ranged from 8 to 19 years (M = 14,9; SD = 3,1), with most of the participants in the 15-17-year-old range (n = 6; 60%). Participants represented a range of diagnoses, including anorexia nervosa, attention deficit hyperactivity disorder (ADHD), autism spectrum disorder (ASD), epilepsy, generalised anxiety disorder (GAD), major depressive disorder (MDD), obsessive-compulsive disorder (OCD), oppositional defiant disorder (ODD), separation anxiety, specific learning disorder, Tourette’s Syndrome and trichotillomania. The majority of parents were mothers (n = 8; 80%), half of which were single parents. One father (n = 1; 10%) participated, and there was one set of mother and father (n = 1; 10%) who participated together.

**Table 1:** Participant demographics

Participant number Current Age	Inpatient Age	Sex	Length of Inpatient Stay	Diagnosis at Inpatient Discharge	Subsequent Additional Diagnosis	Period after Inpatient Discharge	Participant Parent
TS001 8 years	7 years	Male	5 months	ADHD ODD	Attachment Disorder Anxiety	15 months	Mother
TS002 12 years	8 years	Male	6 months	ADHD	ASD OCD MDD GAD	5 years	Mother
TS003 14 years	12 years	Female	9 months	MDD Separation Anxiety Specific Learning Disorder		2 years	Mother and Father
TS004 15 years	12 years	Male	4 months	GAD Trichotillo-mania ASD	Transgender	3 years	Mother
TS005 16 years	12 years	Male	10 months	Anorexia Nervosa Body Dysmorphic Disorder MDD	Social Anxiety	4 years	Father
TS006 19 years	13–14 years	Male	2 months Day patient 1 month	ASD		6 years	Mother
TS007 17 years	10 years	Female	6 weeks	ADHD Specific Learning Disorder OCD Tourette Syndrome	Depression	7 years	Mother
TS008 17 years	9–10 years	Male	1st 4 months 2nd 1 month	ASD Epilepsy Tic Disorder NOS	Tourette Syndrome	8 years	Mother
TS009 16 years	10 years	Male	3 months Day patient 4 months	ASD ADHD		6 years	Mother
TS010 15 years	11 years	Female	5 months	MDD Panic Disorder		4 years	Mother

ADHD = attention deficit hyperactivity disorder; ASD = autism spectrum disorder; GAD = generalised anxiety disorder; MDD = major depressive disorder; NOS = not otherwise specified; OCD = obsessive-compulsive disorder; ODD = oppositional defiant disorder

### Case vignettes to illustrate parent-child perspectives of outcomes after inpatient admission

Our first aim was to describe the experiences of inpatient admission and perceived outcomes after discharge from the inpatient psychiatric admission from the perspective of children and at least one of their primary caregivers, including what they felt helped or hindered their progress after discharge from a pre-adolescent psychiatric inpatient unit in South Africa. Here we will outline five short case vignettes to illustrate some of the feedback. Further details from cases will be presented as part of the thematic analysis and illustrative quotes:

**TS001** – Aged 7 years at the time of admission, interviewed 15 months after discharge. The child was diagnosed with ADHD and ODD and the parent-child dyad reported that short-term improvements were not sustained in the medium-term after discharge. The mother reported improvement both at home and with a facilitator at school. However, while continuing to have follow-up care, his behaviour deteriorated at school 11 months after inpatient discharge.

**TS002** – Aged 8 at admission, interviewed 5 years post-discharge. The child was admitted with a diagnosis of ADHD, and subsequently also diagnosed with ASD, OCD, MDE and GAD. He began attending a new school six months prior to qualitative data collection, having left school a year before following ongoing resistance to attend. He subsequently resumed attending the psychiatric outpatient service, with good effect, according to the mother. Notably, both parents spoke of improvements in their own mental health that occurred prior to recent progress (TS002 and TS004).

**TS004** – Aged 12 on admission, interviewed 3 years post-discharge. The child was diagnosed with ASD and GAD. The child, bullied prior to admission, felt relief moving to a home school. After revealing wanting to change gender a year before this study, psychological care, commenced. Child and parent reported subsequent overall improvement, with the mother delighted by their improved relationship. However, the child was refusing school due to fear of stigma.

**TS008** – Aged 9 at first admission, interviewed 8 years post-discharge. The child was diagnosed with ASD, epilepsy and Tourette’s syndrome. He needed inpatient readmission in his second year post-discharge. Thereafter, becoming a weekday border at his school for autism (which he commenced after his first inpatient admission), was deemed beneficial by both mother and child. The child mentioned having friends at the hostel, albeit younger than him. At the time of qualitative data collection, he was in his final year at the school, and described that he was experiencing frequent panic attacks.

**TS009** – Aged 10 at admission, interviewed 6 years post-discharge. The child became disillusioned, refusing school and his usual follow-up care for a year prior to the qualitative data collection. He and his mother attributed his regression to teachers that were unaccommodating of his ASD diagnosis. His mother also associated the decline with the discontinuation of his Ritalin medication the previous year and stated that he had not made any friends at the secondary school.

### Thematic analysis findings

The themes and subthemes identified in the study are summarised in Table 2. The overarching themes comprehensively delineate the favourable and less favourable experiences of inpatient admission and outcomes at discharge. Overall, two main themes emerged. Theme One (“A turn in the road”) reflected the parent-child dyad’s experience of inpatient admission as a catalyst for positive outcomes. Theme Two (“Still a rough journey”) reflected experiences of ongoing difficulties after discharge.

**Table 2:** Themes and subthemes identified in the study

Theme 1: “A turn in the road” Inpatient admission seen as the catalyst for positive outcomes	Theme 2: “Still a rough journey” Ongoing difficulties after discharge from inpatient admission
**Subtheme 1.1** Improved functioning after inpatient discharge, with knowledge of diagnosis	**Subtheme 2.1** Minimal improvement of children lacking diagnosis
**Subtheme 1.2** Improved functioning after inpatient discharge, when equipped with cognitive and behavioural skills	**Subtheme 2.2** Lingering problems despite improvement
**Subtheme 1.3** Change to appropriate school associated with positive outcomes	**Subtheme 2.3** Regression associated with transition to mainstream secondary school
**Subtheme 1.4** Development of relationships with peers related to positive outcomes	**Subtheme 2.4** Lack of peer relationships associated with negative outcomes
**Subtheme 1.5** Sustained progress associated with continuation of psychiatric follow-up care and medication	**Subtheme 2.5** Negative outcomes associated with discontinuation of psychiatric follow-up care and medication
**Subtheme 1.6** Improved relationships with parents after inpatient discharge, related to positive outcomes	

The majority of families experienced inpatient admission as positive and helpful, and reported positive outcomes, but with ongoing difficulties over time. The two main themes or broad domains emerged from the data as important related to the outcomes of the children after inpatient admission. Diagnostic certainty, newly acquired cognitive and behavioural skills, improved parent-child relationships, appropriate school placements, development of peer relationships, as well as follow-up psychiatric care and medication, were seen as contributing to positive outcomes. Conversely, ongoing difficulties included lingering problems despite improvement, minimal improvement while lacking diagnosis, regression with transition to mainstream secondary school, negative outcomes associated with a lack of peer relationships and discontinuation of psychiatric care and medication. Below we provide further details including illustrative quotes.

### Theme 1: “a turn in the road” - inpatient admission seen as the catalyst for positive outcomes

#### Subtheme 1.1: improved functioning after inpatient discharge, with knowledge of diagnosis

Families described that inpatient admission was instrumental in the diagnosis of their children. For example, parents revealed that two children (aged 10 and 14) were diagnosed with autism spectrum disorders (ASD), and the psychotic episodes of a child with ASD were found to be due to epilepsy. Diagnoses of Tourette Syndrome and obsessive-compulsive disorder (OCD) were made in another child after almost 3 years of outpatient treatment. Other inpatient diagnoses included depressive and anxiety disorders. Parents expressed how diagnosis led to fundamental positive change:

*“I know a lot of people say don’t stick to diagnosis, but it made sense, and everything was so much easier after that”* [Parent 006].

*“It allowed me to understand exactly why I was there, and they helped me work through it”* [Child 006, Male].

*“So this unit saved his life [in reference to a diagnosis that was made]”* [Parent 008].

*“Probably the right medication* (was the thing that helped) *[as a result of a diagnosis being made]”* [Child 008, Male].

#### Subtheme 1.2: improved functioning after inpatient discharge, when equipped with cognitive and behavioural skills

All participant children felt that inpatient admission was beneficial to some extent. The majority learnt coping strategies and social skills. Despite a year of outpatient treatment, one child participant only managed a return to school after inpatient treatment (Child 010, Female). She gained confidence and learnt to manage her anxiety, while another (Child 003, Female) was helped when empowered to express herself constructively. This is evident in the following quotes:

*“I won’t say she was her confident old self that she was before she became anxious, but you could see she was more settled, more calm, she didn’t get anxiety attacks anymore, because they taught her how to control it”* [Parent 010].

*“I went from this scared person, scared person that was drawing away from… in her own space, to being a person who was open and talked to everyone”* [Child 010, Female].

*“And I think if it wasn’t for the admission [making reference to the skills acquired through the admission]… it would have been a very different outcome”* [Parent 006].

*“The best part… being able to fold my clothes, just getting ready for bed, and easy, I think I enjoyed that. That gave me the time to realise okay this is what I’m doing, and this is how I can apply it to real life”* [Child 006, Male].

#### Subtheme 1.3: change to appropriate school associated with positive outcomes

All children were appreciative of change to an accommodating educational environment. The three who remained at an appropriate school (Child 003, Female; Child 006, Male; Child 010, Female), continued to succeed. The participant who changed to a school for autism (Child 008, Male), learnt cognitive and behavioural skills there, whilst his parent felt supported which highlighted the importance of collaborative efforts between schools and parents, which outlines the significance of support systems to foster a positive and inclusive educational environment for both children and their parents. Participants said:

*“That changed his life 100%, because he would be very different now, I don’t think he would have finished school to be honest”* [Parent 006].

“[going to an appropriate educational environment was] *… exactly same thing that I did here* [in the inpatient unit], *just a bigger scale”* [Child 006, Male].

“[School for autism] *is now our family, next year it’s [Child 008] and I, totally in the real world alone”* [Parent 008].

*“I probably wouldn’t be at the school I am now, and without that I probably wouldn’t have finished school in itself”* [Child 008, Male].

#### Subtheme 1.4: development of relationships with peers related to positive outcomes

Regardless of diagnosis, the development of friendships was associated with positive outcomes. Satisfaction with friendships was reported by all children with sustained improvement. This is indicated in the following comments:

“*It gave him the ability to make friends outside”* [Parent 009].

*“Some of the best time I’ve ever had…* making friends at my new school” [Child 009, Male].

*“And he would always joke and say because everybody’s abnormal like him at* [the appropriate educational environment] *… so he became a different person”* [Parent 006].

“*Partying and stuff, I just never saw that. I always thought I’d be a book guy forever, and then next thing you know I was like, made friends”* [Child 006, Male].

#### Subtheme 1.5: sustained progress associated with continuation of psychiatric follow-up care and medication

All children with positive outcomes still attended outpatient follow-up and still required medication. The quotes below describes how both parents and children view the outpatient care and follow-up as helpful to their experience and how a difference in progress can be seen, as suggested by the following:

*“Just for seeing her from where she was, and now where she is, I must say, it’s like a star shining in the sky”* [Parent 003].

*“It got much better. There’s still ups and downs, but not a lot”* [Child 003, Female].

*“It’s just thankfulness … that’s how I feel when I walk in here [referring to the outpatient wing of preadolescent unit] ”* [Parent 010].

*“I believe everyone has a point in time when they need help, and um, for me I have my mom, and I have God, and I have the doctors here [referring to the outpatient wing of preadolescent unit] that I see every 6 months”* [Child 010, Female].

#### Subtheme 1.6: improved relationships with parents after inpatient discharge related to positive outcomes

Parents commented on the marked reduction of conflict at home. Two children (Child 003, Female; Child 010, Female) were enabled to communicate their concerns to parents and found this helpful. This is illustrated in the following quotations:

*“We no longer had the constant fighting … My home was a battlefield, it was like a warzone”* [Parent 009].

*“Helped with coping with emotions”* [Child 009, Male].

*“And she’ll tell me if something made her unhappy, which she never did”* [Parent 010].

“*Learnt to speak about difficulties and to voice opinion”* [Child 010, Female].

### Theme 2: “still a rough journey” - ongoing difficulties after discharge from inpatient admission

#### Subtheme 2.1: minimal improvement of children when diagnosis lacking

Participant parents claimed inpatient treatment was largely ineffective for the child who received subsequent diagnoses of ASD, OCD, generalised anxiety disorder (GAD) and major depressive disorder (MDD) (Child 002, Male), and the child discovered to be transgender (Child 004, Female). However, both children claimed it lowered their aggression. Incongruent experiences between parents and children can be seen, where the disparity can be noted in the reports by participants, which foregrounds the value of parent-child dyads in understanding the experiences and outcomes:

*“I got worse, he got worse, he wasn’t better when he came out”* [Parent 002].

*“Did help slightly with my behaviour and everything… my physical violence was lowered quite a bit”* [Child 002, Male].

*“So I wouldn’t say anything improved hugely”* [Parent 004].

*“I felt a lot better, I was definitely calmer”* [Child 004, Male].

#### Subtheme 2.2: lingering problems despite improvement

One participant (Child 001, Male) required additional medication and therapy in the medium-term outcome period, while four encountered setbacks in the long-term (Child 005, Male; Child 007, Female; Child 008, Male; Child 009, Male). Symptoms remained evident in participants with ASD (Child 008, Male; Child 009, Male), although no symptoms were mentioned for one participant (Child 006, Male). Participants commented:

*“Anxiety has obviously remained a challenge”* [Parent 008].

*“And then just went up and down up and down, until now*” [Child 008, Male].

*“I thought it had been resolved… and there was nothing, the scars were fading and then I checked again and there she was cutting again”* [Parent 007].

*“There was a stage where I really felt overwhelmed, and I was harming myself, I don’t do that anymore though”* [Child 007, Female].

#### Subtheme 2.3: regression associated with transition to mainstream secondary school

A participant (Child 009, Male) who had received a diagnosis of ASD managed well in a new mainstream primary school, but not after the transition to secondary school. A participant (Child 007, Female) who flourished after starting in an inclusive class at a new primary school, needed to resume psychiatric treatment after transitioning to a mainstream secondary school. A third child (Child 005, Male) who changed secondary school and stopped having a private tutor and classroom, struggled to cope.

“*Junior school was fantastic; High school crash and burn”* [Parent 009].

*“Uh, so I mean another teacher that was another clash of personality was my grade 8 and grade 9 math teacher… it made maths for me hard, which previously was actually quite easy…”* [Child 009, Male].

*“Um, she’s had some very traumatic things happen to her at the high school…”* [Parent 007].

*“I realised I had a lot more responsibilities, and a lot more work to do”* [Child 007, Female].

#### Subtheme 2.4: lack of peer relationships associated with negative outcomes

For all child participants with negative outcomes, a lack of friends was reported. Loss of peer relationships was evident for those who deteriorated with change of school. Participants expressed:

*“He hasn’t made many friends, he finds it difficult to socialise”* [Parent 005].

*“I listen to music, but I’m not doing much else”* [Child 005, Male].

*“He still struggles. That’s part of his isolation at school, he’s totally isolated… he has his old friend back now”* [Parent 002].

*“I’ve changed so much. I don’t really relate to very many people”* [Child 002, Male].

#### Subtheme 2.5: negative outcomes associated with discontinuation of psychiatric follow-up care and medication

Poor outcome was associated with the two participants who discontinued post-discharge follow-up (Child 002, Male; Child 004, Male). Moreover, deterioration resulted in two 16-year-old participants refusing to continue follow-up care, although one child (Child 005, Male) continued taking medication. The other (Child 009, Male) had also discontinued school the previous year, which had followed his discontinuation of Ritalin (stimulant medication prescribed for ADHD) the year prior. Participants said:

*“I took him out of school, because again, everything with him, was just falling to pieces”* [Parent 002].

*“So it’s been still kind of bad… but I think now it’s okay*” [Child 002, Male].

*“When he went off Ritalin that’s when everything, he just crashed”* [Parent 009].

*“When you realise that you don’t know what you want to do with your life… and when you can’t find something, it sort of demotivates you, and I, at the moment I’m very demotivated”* [Child 009, Male].

## Discussion

Inpatient admission may represent an important component of psychiatric care for pre-adolescents with complex, severe or pervasive mental health disorders. However, research on outcomes after admission of pre-adolescents to date has been remarkably limited and of variable quality. This study therefore aimed to describe parental and child perspectives about their experience of inpatient psychiatric admission and outcomes after discharge from a pre-adolescent unit, and to use these experiential data in thematic analysis to identify potential outcome variables that would be meaningful to families. We used a qualitative methodology and semi-structured in-depth interviews of ten parent-child dyads (a) to describe perspectives of ten children who have had a pre-adolescent inpatient psychiatric admission in Cape Town, South Africa and of one or more parent, and (b) to analyse these experiential data using thematic analysis. Our goal was to identify outcome variables of importance to families and service users that could be used in future outcomes-based research.

The study participants largely identified as male between the ages of 7 and 14 years of age during their inpatient psychiatric admission between 2012 and 2017. The children had a range of psychiatric and neurodevelopmental disorders. Participants were interviewed between 15 months and 8 years post-discharge. Half of the participants described the helpfulness of the new intervention techniques they were taught (e.g. cognitive and behavioural techniques) and associated the skills development with positive outcomes. However, families also described that they had, almost without exception, ongoing challenges. Thematic analysis identified two broad themes – the first, positive theme indicating that inpatient admission was experienced as life-changing and positive by participants; the second theme captured the ongoing difficulties experienced in family journeys with mental health disorders. These were captured by the powerful quote “A turn in the road, but still a rough journey”.

The finding of positive perspective about admission is similar to results from parent training programmes [19]. Parents remarked how these acquired skills resulted in an improved relationship with their child. Short-term studies that emphasised the inclusion of parents in therapeutic work, reported similar success [20, 21]. In our qualitative data, short-term improvements were not sustained in the medium-term (e.g. TS001) where the mother reported improvement both at home and with a facilitator at school in the short-term. However, while continuing to have follow-up care, her child’s behaviour deteriorated 11 months after inpatient discharge. This finding was very much in keeping with short to medium-term outcomes, where improvement was mostly found at five- and six-months post-discharge in previous studies [22–24]. The case vignette of TS008 (a child who needed inpatient readmission in his second year post-discharge) was in line with the existing literature of medium-to-long-term outcomes, for example a study [25] that found sustained improvement one year post-discharge, but negative changes in outcome within two years reported in an earlier review by Blotcky and colleagues [11, 25]. Ongoing problems were found three years post-discharge in previous studies [24, 26]. Interestingly, no ongoing problems were reported in our qualitative data for the oldest participant (19 years old) diagnosed with ASD (Child 006, 6 years post-discharge), although he was taking anti-anxiety medication in the year of data collection, after completion of secondary school. A further two parent-child dyads (Child 009, 6 years post-discharge; Child 007, 7 years post-discharge) described a largely positive outcome until entering mainstream secondary school two years prior to qualitative data collection. All the participants had changed to more appropriate educational environments soon after inpatient discharge, viewed as fundamental to their positive progress by families (Child 003, Female; Child 006, Male; Child 007, Female; Child 008, Male; Child 009, Male; Child 010, Female). Likewise, an identified 16-year outcome study [27] associated a change of school with positive outcomes.

This thematic analysis revealed that parent-child dyads highly valued the diagnostic certainty and skills obtained during inpatient admission (subtheme 1). It also exposed factors seemingly necessary for positive long-term outcome. These largely mirror those identified in a study of child and adolescent psychiatric inpatients: the support of family, friends, school staff and mental health professionals, including coaching in social skills [28] and acquisition of cognitive and behavioural skills (subtheme 2). Ignorance of CAMH problems is an obstacle to receiving professional help [29]. Our study highlighted the contribution that an appropriate educational environment (subtheme 3) makes towards positive mental health outcomes, particularly as that was where participants made friends (subtheme 4). In order to succeed in an educational environment, cognitive and behavioural engagement have been found necessary alongside feeling emotionally content [30]. The appropriate school placement for children and adolescents fosters a sense of community and safety, as seen in the study, thus creating a conducive environment for the well-being and academic progress of the students. Schools have been identified to be powerful environments for mental health programmes [31, 32], which have been found to increase community understanding and result in less school drop-outs. Preyde and colleagues [28] reported that child and adolescent inpatients had ‘overwhelming’ concerns about engaging in the school environment while having mental health difficulties. Apart from worrying about coping with the academic aspect of school, most feared social isolation, or loneliness. Comprehending and attending to these concerns is vital to helping these youths attain a positive trajectory into the future [28]. Social interventions, such as school adjustments and inclusive schooling, have a significant impact on the outcomes for children after inpatient psychiatric admission. While inpatient admission does not provide all the positive outcomes it is essential that long-term support outside the psychiatric unit is equally as important. Examples of approaches and social interventions to ensure ongoing support could include individualised education plans, teacher training, peer support programmes, parental involvement, community partnerships, and addressing stigma. The continuity of care through social interventions and long-term monitoring is important for the successful reintegration of children and adolescents into the school environment. These interventions provide a supportive and inclusive educational setting, which contribute to improved well-being for children even after discharge from inpatient admission.

All participants who expressed positive outcomes post-discharge had continued follow-up care (subtheme 5) and where medications were stopped (for example case vignette TS009), outcome was worse. These findings are in line with most earlier outcome studies [11, 13, 33, 34]. Subtheme 6 (improved relationships with parents related to positive outcomes) was another important variable that emerged from the thematic analysis. In case vignette TS003, the mother powerfully commented on the impact of an improved parent-child relationship on outcome. Not identified as a specific subtheme, but related, parents also spoke of improvements in their own mental health that was associated with improvements in their child.

Overall, inpatient psychiatric admission was viewed as a ‘life-changing’ contributor to the experiences and outcomes by most parent-child dyads, summarised by the theme “a turn in the road” and was associated with positive long-term outcomes by more than half of parent-child dyads. However, many families commented on ongoing challenges, as summarised by the theme “but still a rough journey”. These themes suggest both the favourable and less favourable experiences and outcomes of inpatient psychiatric admission. Some differences in the opinions of parents and children could be seen in the second theme of the study. These differences about the experiences and outcomes of inpatient psychiatric admission highlight the potential value of conducting parent-child dyad studies, allowing an understanding of the nuances of both parental and children’s experiences and communication dynamics. We believe that inclusion of parents and children therefore provided a more comprehensive and holistic picture of the experiences and outcomes of inpatient psychiatric admission. To our knowledge, no inpatient outcome study to date has used this methodological approach.

### Study limitations

We acknowledge that this qualitative study presented data from only ten parent-child dyads who had attended one pre-adolescent inpatient mental health unit in Cape Town, South Africa. We can therefore not presume that findings may be generalisable to other units in South Africa, Africa or internationally. However, to our knowledge, this is the first ever qualitative study to explore parent and child perspectives about outcomes related to inpatient mental health admissions. From this perspective, findings may at least stimulate further exploration of similar functional outcomes in other clinical settings and in future research. We further acknowledge that, apart from the small sample, follow-up periods were not very long for the majority. However, a strength of the study design was the parent-child dyadic data that provided complementary findings from both perspectives. As outlined in the methods, we recognise that qualitative analysis posed risks of biases. We have tried to increase the trustworthiness and reflexivity of findings through the use of independent data collectors, and the inclusion of a senior, independent co-rater. We acknowledge that the associations described here do not imply any quantitative statistical correlations but rather the association within the narratives shared by the participant dyads.

## Conclusions

In this qualitative study of parent-child dyad perspectives about outcomes after inpatient mental health admission for pre-adolescents, the majority of families found inpatient admission positive and helpful, and indicated positive outcomes – but with ongoing difficulties over time. The 10 parent-child dyads identified diagnostic certainty, school placements, friendships and ongoing psychiatric follow-up as important factors contributing to outcomes. It is therefore noteworthy that parent and child perspectives were much more focussed on functional outcomes (such as participation in school and community activities), than in measurable mental health/behavioural outcomes (as those typically used in outcome studies). In addition, an accommodating educational environment seemed to be a vital contributor to positive mental health outcomes for children and adolescents, particularly as an environment for the establishment and maintenance of friendships. The concerns expressed about the detrimental impact of mainstream secondary schools on the mental health of young people underline the importance of also considering mental health expertise, knowledge and interventions in schools, and not only in health-based contexts.

## Data Availability

The dataset used and/or analysed during the current study is available from the corresponding author on reasonable request.
